# App-Based Tele Ophthalmology: A Novel Method of Rural Eye Care Delivery Connecting Tertiary Eye Care Center and Vision Centers in India

**DOI:** 10.1155/2019/8107064

**Published:** 2019-12-18

**Authors:** Anthony Vipin Das, Shravani Mididoddi, Priyanka Kammari, Navya Deepthi Davara, Abhinav Loomba, Rohit Khanna, Mukesh Taneja

**Affiliations:** ^1^Department of eyeSmart EMR & AEye, L V Prasad Eye Institute, Hyderabad, India; ^2^Department of Teleophthalmology, L V Prasad Eye Institute, Hyderabad, India; ^3^Department of Cornea & Anterior Segment, L V Prasad Eye Institute, Hyderabad, India; ^4^Gullapalli Pratibha Rao International Center for Advancement of Rural Eye Care, L V Prasad Eye Institute, Hyderabad, Telangana, India

## Abstract

**Purpose:**

The study aims to describe a novel method of utilization of the eyeSmart EMR (electronic medical record) app. It describes the demographic distribution, clinical presentation, query requested by the vision technician, and management advised to the patients by using “eyeSmart EMR” app from the vision centers located across a three-tier eye care network in India.

**Methods:**

This is a retrospective review of all patients who required a tele-ophthalmology consultation from January 2017 to August 2018. The demographic, clinical details, and the impact of teleophthalmology using eyeSmart app, in the vision centers of a three-tier eye care network, was analyzed in detail.

**Results:**

A total of 15,001 tele-ophthalmology consults were included which is from January 2017 to August 2018. The mean age was 38 ± 19 years and male to female ratio was 1 : 2. Video calls were performed for 6191 (41.27%) consults and the impact was measured. Additional clinical information was received in 65.61% consults through video call. Medical management was advised in 47.07% of patients and 30.30% were referred to higher centers for medical intervention and 0.59% were referred for surgical intervention, 16.23% were prescribed glasses. No intervention required for 0.69% of cases. Hence nil intervention was advised.

**Conclusion:**

The combination of using tablet and video calls with the help of eyeSmart EMR app is a novel method in teleophthalmology. It helps in connecting the patients at rural areas and the ophthalmologists in higher centers. The use of technology plays a vital role in the appropriate medical management of the patient.

## 1. Introduction

Teleophthalmology is a branch of telemedicine which aims to increase access to eye care for remote and rural populations across the world. There are various forms in tele ophthalmology which are aimed in treating the patients with different ocular diseases. Smart phones and tablets are currently used to gather the ocular images of the patient and can be shared with the ophthalmologist for further diagnosis and medical management. Boissin et al. compared the quality of image between the laptop or computer screen and smart phone and reported that the smart phone screens can be substituted with the computer or laptop screens for the detection of ocular diagnosis through a quality image [1]. Blackwell et al. reported that the ophthalmology is suitable for treating the patients using telemedicine. They reported that both patients and staff are satisfied with the telemedicine services as it offers useful benefits for the patients and also enhances the skill sets of local ophthalmologists [2]. Majority of these teleophthalmology services are through asynchronous methods (i.e., store-and-forward of images), some use a hybrid of real-time and store-and-forward methods and fewer used synchronous methods (e.g., Video conferencing) [3]. Maa et al. reported the outcomes of patient satisfaction (4.95 out of 5), disease detection, eyeglass remakes (0.59%), based on the technology-based eye care services (TECS) in 5 primary eye care centers in Georgia [4]. For a wider outreach, teleophthalmology is gaining interest as it contributes to saving time and money involved in travel to seek an opinion of an ophthalmologist [5–9]. In India, teleophthalmology consultations hold great potential to reach the remote rural populations to enable access to eye care due to challenges in distance and access to quality eye care. This study aims to describe a novel method of utilization of the eyeSmart EMR (electronic medical record) app to capture the demographic distribution, clinical presentation, query requested by the vision technician and management advised across 173 vision centers located across a three-tier eye care network in India.

## 2. Methods

EyeSmart EMR is an in-house electronic medical record system implemented across the LV Prasad Eye Institute (LVPEI) network and has enabled 4.8 million consultations since its inception in August 2010. EyeSmart EMR app was launched in the year 2016 to digitize the 173 rural vision centers across the LVPEI network and the app has enabled over 501,771 consultations for rural patients. Tele-ophthalmology and video calling are additional services provided through eyeSmart EMR system.

LVPEI's eye care network provides its services at different levels of the eye care system in the 4 states (Andhra Pradesh, Telangana, Karnataka, and Odisha) of India. It constitutes 1 centre of excellence, 3 tertiary centers, and 18 secondary centers. Each of these secondary centers have 10 vision centers connected to them to refer patients for higher medical or surgical care (total *N* = 173). Each vision center serves a rural population of 50,000 individuals. The vision center is manned by a single vision technician who is trained on the basic clinical tests which are essential for a basic eye examination at the centre of excellence for a period of 1 year. Every vision center is equipped to deliver primary eye care services such as vision testing, spectacle prescription, and slit lamp examination. The eyeSmart EMR app was installed on an android tablet (iBall Slide Brace XJ) and connected to the slitlamp biomicroscope (Carl Zeiss SL 115). The app enables capturing the demographic, clinical information and images (anterior segment) of the eye for a teleophthalmology consultation through the cloud. The internet connectivity was achieved on the tablet through a 3G network SIM card (Idea 3G prepaid). The video conferencing tool of Skype was used for all the teleophthalmology consults (Skype, Microsoft Corp, Redmond, USA).

**Figure 1:**
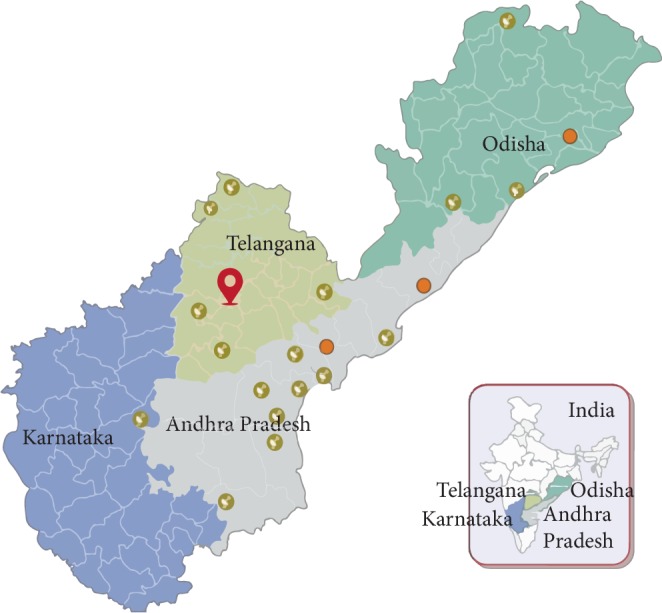
Graphical representation of VC across the network. Note: The red colored location symbol denotes the command center, which was located at the center of excellence and from where all the teleconsultations were advised. The green colored circles are the secondary centers. Vision centers were connected to the respective secondary centers.

All the teleophthalmology consultations requested from 173 vision centers across the LVPEI network are received at a command center stationed in the center of excellence (Figure 1).The patient demographic details are registered by the vision technician at the respective vision center through the tablet by using the eyeSmart EMR app. The preliminary examination is then performed, which includes history taking, such as chief complaint, present and past illness, systemic history, family history, previous surgical history, and then general examination, the visual acuity recording, objective and subjective refraction and final glass prescription. The slit lamp examination is then performed and then followed by capturing of the images of the eye (anterior segment) as relevant using the tablet attached to the eye piece. The ocular images (anterior segment) are then synced online through the app for an ophthalmologist opinion through the EMR. Information related to the ocular condition or a query is sent to the command center by the vision technician after examining the patient at the vision center. Queries would include the establishment of an ocular diagnosis, medical management, and surgical management, training purpose (in which they can ask for findings from the case or any differential diagnosis) and whether to refer to a higher centre.

**Figure 2:**
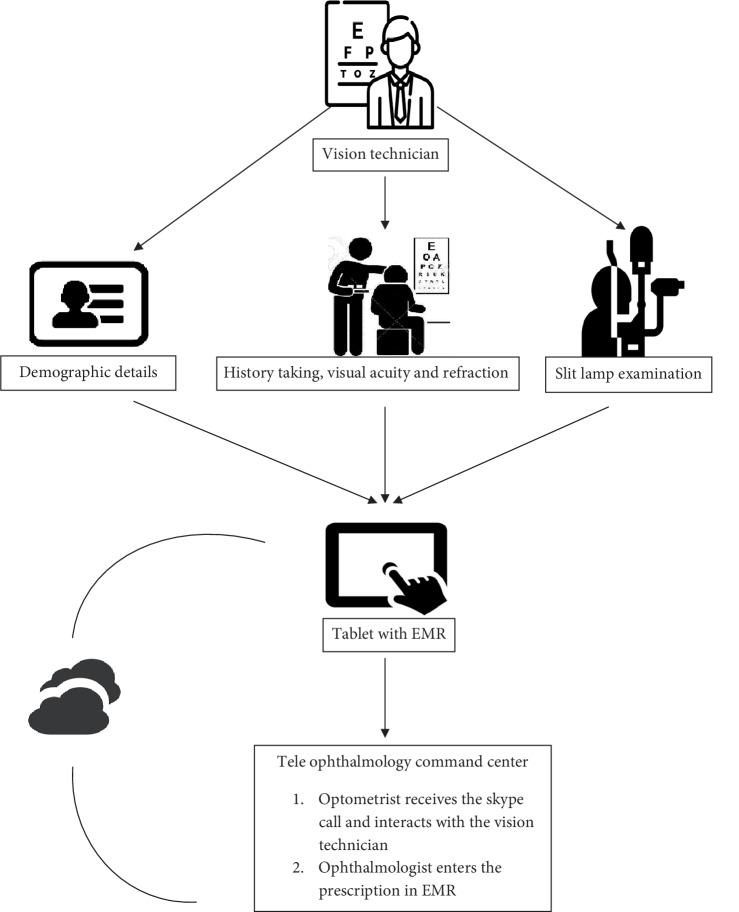
Steps involved in teleophthalmology consultation.

The teleophthalmology command center is situated at the center of excellence which receives the teleconsultation request in real time and has access to the eyeSmart EMR app of all the vision centers across the network. The vision technician connects the patient to the ophthalmologist present at the command center through a video call using Skype services available in the tablet. The ophthalmologist at the command center reviews the clinical information and images and diagnose the ocular condition. The nature of the disease and the possible interventions (medical or surgical) are discussed with the patient through a Skype call by the Ophthalmologist. The patient is then referred to the secondary or tertiary eye care center through eyeSmart EMR app, for further medical or surgical management as needed. The advice given by the ophthalmologist is synced via the cloud to the eyeSmart EMR app for documentation. After every teleophthalmology consultation, the ophthalmologist at the command center selects the following options as relevant to measure the impact of teleophthalmology i.e., (i) you gained more information from the patient with the video call, (ii) you advised medical/surgical consult at higher center, (iii) you prescribed medical management to the patient, (iv) the tele consult was not helpful. The impact of using tablets accompanied by a slit lamp photograph and video call in treating patients is measured through the responses given. An overview of the process of teleophthalmology consultation using the eyeSmart EMR app is described in Figure 2.

This was a retrospective study, which included all the patients, who visited our vision centers of the LVPEI network during January 2017–August 2018 in whom teleophthalmology consultation is required. Patients who did not require a teleophthalmology consultation were excluded. The collected variables from the teleophthalmology were age, gender, geographic locations, chief complaint, query requested, teleophthalmology advice given, and the impact of Skype video call. Data of the patients were collected from the EMR database of LVPEI and analyzed by using Microsoft Excel-version 2010.

## 3. Results

A total of 15001 teleconsultations were seen from July 2017 to August 2018 across the LVPEI network. Mean age of all the patients was 38 ± 19 years. Majority of these patients were males (*N* = 9347) compared to females (*N* = 5654). Most of the patients belong to Andhra Pradesh (*N* = 8792; 59%) followed by Telangana state (*N* = 5999; 40%) (Table 1).

**Figure 3:**
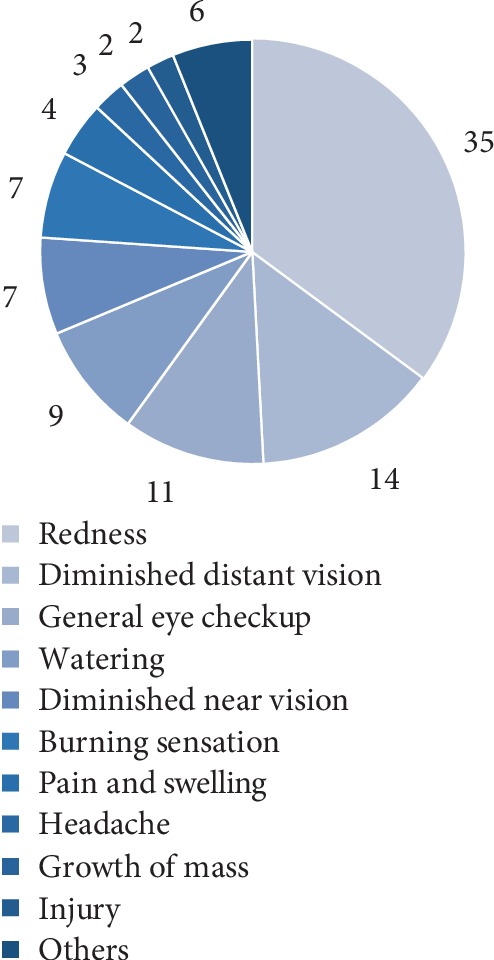
Distribution of complaints with which patients walked into the vision center.

The presenting chief complaints of the patient at the vision centers were categorized into 11 different categories and the details are shown in Figure 3. The patients who reported to have redness as their chief complaint was highest (36%) and in very few patients (1%) had pain as their chief complaint. The query requested by the vision technician from the vision centers were categorized into 6 different types of queries, i.e., query for diagnosis, query for infection, query for medical management, referral for surgery or referral for dilated fundus examination and for training purposes, the details of each query was shown in Table 2.

Query for diagnosis (*N* = 8059; 54%) indicates that the provisional diagnosis made by the vision technician needs to be confirmed through the tele request by an expert from the command center and this is the most common query of all. The second and third most commonly requested queries were “query for medical management” (*N* = 2996; 19.97%) and “query for surgery” (*N* = 2476; 16.51%), respectively. Query for dilated fundus examination was sent for very few patients (*N* = 15; 0.10%). Queries requested to rule out any infection by seeing the image were in 1339 (8.93%) patients. Although many queries were sent to make diagnosis, few of them were treated medically and others such as microbial keratitis, injury cases and cases which need dilated fundus evaluation were referred to higher centers.

**Figure 4:**
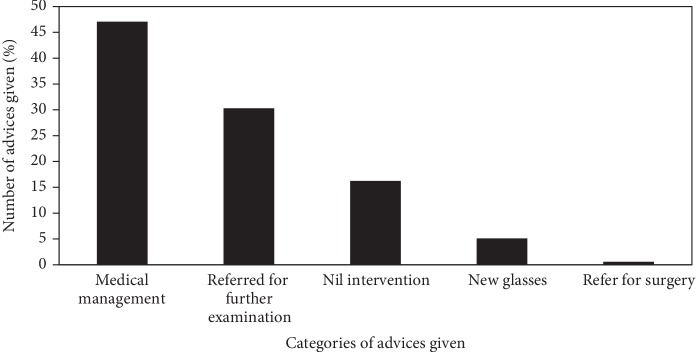
Distribution of categories of advices given to the patients at vision centers.

The advice given from the command center in return to each of the requested queries were categorized as medical management, referral to the higher center who may need any investigations, dilated fundus examination, referral for surgical management, new glasses, and nil intervention. The same advice will be reflected at the vision center in the eyeSmart EMR app. A significant number of teleophthalmology consults were treated medically (47.07%) followed by further management (30.30%) and least were referred for surgical management (0.59%) (Figure 4).

**Figure 5:**
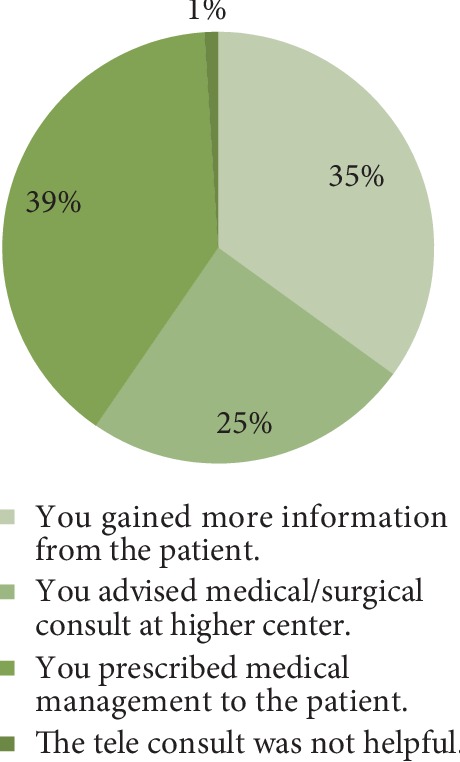
Distribution of impact through video call.

Of the total of 15001 teleophthalmology consultations, 6191 consults had video calls for patient's extra information. To know the impact of skype call, few options were given to the ophthalmologist at command center. It shows that, by using Skype teleconsultation, 4576 (39%) consultations were medically managed and 2860 (25%) patients were referred to higher centers for medical or surgical intervention. The video call was not helpful in 118 (1%) cases. The distribution of impact shown through video call was shown in Figure 5.

## 4. Discussion

This study describes the utilization of eyeSmart EMR app for teleophthalmology consultations through video conferencing across the vision centers of LVPEI eye care network in India. The highest teleconsultations were performed on the patients belonging to Andhra Pradesh state followed by Telangana, of which the majority were males. The common approach in the field of teleophthalmology is to capture still or video images of the patient, where the images are acquired by a technician and sent to a different location for diagnosis and plan of management [10]. Boisin et al. reported that the images captured from the smart phones or tablets can be utilized for the disease detection in place of computer or laptop screens due to the quality of the image displayed from these screens. However, the reliability of diagnosing the disease was lacking in this study as the focus of the study was on grading the image quality rather than how far it was helpful in diagnosing a particular disease [1]. Kumar et al. reported that the tele-ophthalmology services are cost-effective for the patients in the rural areas in the substitute of conventional eye care at the higher centers [11]. Real time video transmission using tablet attachment in teleophthalmology has recently been described in literature [12]. Similar to these two studies, 73% of the patients in the rural centers could receive timely intervention in the current study with the help of eyeSmart EMR app in a tablet which had a verbal and video facility for face to face interaction of the patient and ophthalmologist occurred through the command center. However, the remaining few patients were referred to higher centers for further management. Caffery et al. reported that, from the 62 different models of teleophthalmology in eye care in 19 different countries, the most prevalent services were comprehensive eye examination (*N* = 17; 26%) and emergency eyecare services (*N* = 8; 12%).The disease-specific teleophthalmology services were most commonly performed to diagnose diabetic retinopathy and glaucoma conditions [3]. In most of the studies the ocular images were captured by the available local clinician and transmitted to an ophthalmologist for further evaluation, but in the current study, the ocular images were captured by the well-trained vision technician who plays a vital role in diagnosing various ocular conditions. Along with high-quality ocular image transmission, a one to one real-time interaction was possible through Skype facility in the tablet. Similar to other studies 53.72% of the requested queries through teleophthalmology services in the present study were to detect the diagnosis of a particular disease.

## 5. Conclusion

Teleophthalmology through eyeSmart EMR helped in attending the patients on live basis. It helped patients to talk to the ophthalmologist on lively basis irrespective of the distance they are from. All the patients were given appropriate treatment based on the ocular condition. It also helped to refer the patients appropriately to the respective centers where teritiary eye care services are available. However, challenges of connectivity and power in the rural areas can be overcome with simple high speed internet connections, power backup facilities. However, further studies are required to conclude about the financial benefits and actual impact of tele-ophthalmology services in remote areas.

## Figures and Tables

**Table 1:** Number of patients seen in different states.

State	*N*	*N*%
Andhra Pradesh	8792	58.61
Telangana	5999	39.99
Odisha	179	1.19
Karnataka	31	0.21

**Table 2:** Distribution of queries sent.

Query requested	*N*	*N*%
Query for diagnosis	8059	53.72
Query for medical management	2996	19.97
Query for surgery referral	2476	16.51
Query for infection	1339	8.93
Query for training	116	0.77
Refer for dilated fundus examination	15	0.10

## Data Availability

Corresponding author will be responsible to provide the data as per the need.
